# Osteochondroma of the Distal Volar Thumb

**Published:** 2019-09-30

**Authors:** John Chao, Dieter Brummund, Ramazi Datiashvilli

**Affiliations:** ^a^Division of Plastic and Reconstructive Surgery, Department of Surgery, Rutgers/New Jersey Medical School, Newark; ^b^St George's University School of Medicine, West Indies, Grenada

**Keywords:** osteochondroma, exostosis, thumb mass, hand tumor, bone tumor

## CASE DESCRIPTION

A 48-year-old right-hand-dominant, otherwise healthy, woman presented with a right thumb volar boney mass. The mass had progressively enlarged over 18 years and significantly affected physical use of her thumb. On examination, she had a 2-cm firm boney mass on the volar distal thumb phalanx. She had static 2-point discrimination of less than 6 mm and good distal capillary refill. Radiographs revealed pedunculated, well corticoid, ossific density of the distal phalanx consistent with an osteochondroma. The mass was surgically removed.

## QUESTIONS

1. How often do osteochondromas involve the hand and which bones are most affected?
2. How are osteochondromas of the hand diagnosed?
3. What is the differential diagnosis of osteochondroma of the hand?
4. What is the management of bony hand tumors?

## DISCUSSION

Osteochondroma, also known as exostosis, are cartilage-capped boney outgrowths that occur most often at the metaphysis of long bones or areas of tendon insertion.^1^^,^^2^ Bones most often implicated are the femur > humerus > tibia. Involvement of flap bones (ilium, scapula) may occur, but small-bone involvement of hand, feet, ribs, and vertebrae is rare.^3^ Osteochondromas are the most common cartilaginous tumors, but in the hand, they are less common than enchondromas. Osteochondromas of the hand are primarily found at the distal proximal phalanx and grow outward away from the joint.^1^^,^^2^^,^^4^ Whether sporadic or associated with multiple hereditary exostosis, pathogenesis is associated with *EXT1* and *EXT2* mutations.^5^^,^^6^ There is a 0.5% to 1% chance of malignant degeneration to chondrosarcoma.

Patients typically present with complaints of limited motion of the finger or a bothersome mass.^7^ Initial evaluation includes a thorough physical examination of the hand with a focus on the integrity of the surrounding bone, soft tissue, tendons, nerves, and vasculature. Hand radiographs are key to initial assessment, followed by computed tomography or magnetic resonance imaging if local invasion of the surrounding bone or soft tissues is suspected.^1^^,^^2^ A typical radiograph will show a pedunculated bony stalk continuous with the cortex, with the cancellous bone of the stalk communicating with that of the underlying bone.^3^ Definitive diagnosis requires histological studies from excised or biopsied tissues.^4^

Differential diagnosis includes periostitis ossificans, bizarre paraosteal osteochondromatous proliferation (Nora's lesion), and Turret exostosis, which have all been proposed to be variants of a lesional spectrum.^7^^,^^8^ Paraosteal sarcoma and chondrosarcoma are additional differential diagnoses featuring a more aggressive course. Radiographs are key to distinguishing between osteochondromas, which are continuous with the medullary canal of the underlying bone, and Nora's lesion and paraosteal sarcomas, which are not.^1^^,^^2^^,^^8^

Asymptomatic and stable osteochondromas may be observed; however, treatment is indicated in the presence of local irritation, deformity, a cartilaginous cap of more than 2 cm, or a new onset of growth and symptoms in adulthood. Treatment involves intralesional excision and curettage to preserve stability without decortication of the underlying bone. If a more aggressive variant such as Nora's lesion is suspected, en bloc resection including the capsule, underlying periosteum, and decortication of any abnormal underlying host bone has been shown to reduce risk of recurrence.^2^^,^^8^

Osteochondromas rarely involve the hand or small bones. Initial evaluation involves assessment of surrounding structures and radiographic imaging. Patients typically present with limited motion of the finger. Excisional treatment is indicated in the symptomatic patient.

## Figures and Tables

**Figure 1:**
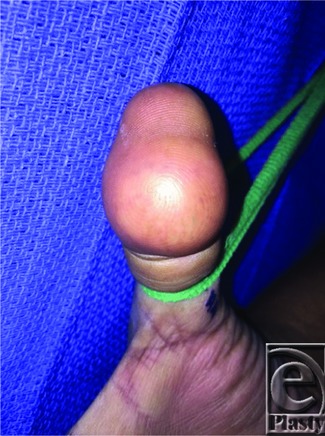
Preoperative photo showing volar thumb mass.

**Figure 2:**
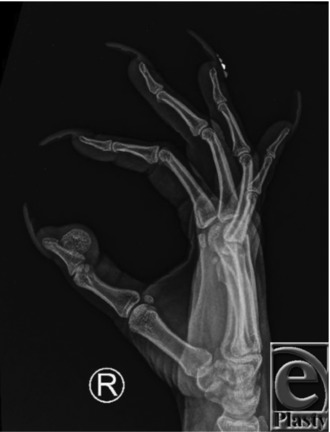
Preoperative x-ray showing pedunculated mass off volar proximal aspect of distal phalanx.

**Figure 3:**
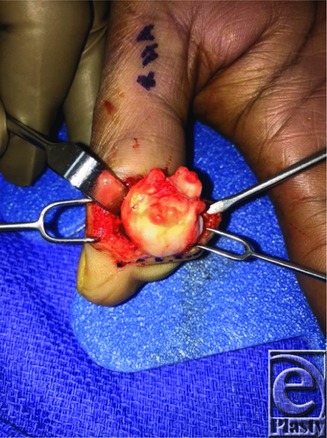
Intraoperative photo of well circumscribed bony mass.

## References

[1] Henderson M, Neumeister MW, Bueno RA (2014). Hand tumors: II. Benign and malignant bone tumors of the hand. Plast Reconstr Surg.

[2] Ward RA, Crosby MA, Janis JE (2017). Benign and malignant masses of the hand. Essentials of Plastic Surgery.

[3] Mantilla JG Osteochondroma. http://www.pathologyoutlines.com/topic/boneosteochondroma.html.

[4] Besser E, Roessner A, Brug E, Erlemann R, Timm C, Gundmann E (1987). Bone tumors of the hand: a review of 300 cases documented in the Westphalian Bone Tumor Register. Arch Orthop Trauma Surg.

[5] Jones KB, Morcuende JA (2003). Of hedgehogs and hereditary bone tumors: re-examination of the pathogenesis of osteochondromas. Iowa Orthop J.

[6] Yuen M, Friedmann L, Orr W (1992). Proliferative periosteal processes of phalanges; a unitary hypothesis. Skeletal Radiol.

[7] Stahl S, Schapira D, Nahir AM (2000). Turret exostosis of the phalanges presenting as limited motion of the finger. Eur J Plast Surg.

[8] Michelsen H, Abramovici L, Steiner G (2004). Bizarre paraosteal osteochondromatous proliferation (Nora's lesion) in the hand. J Hand Surg Am.

